# Influence of student’s ability to delay gratification on their educational transition choice

**DOI:** 10.1186/s40461-022-00134-6

**Published:** 2022-05-13

**Authors:** Gowhar Rashid Ganie

**Affiliations:** Department of Educational Planning, National Institute of Educational Planning and Administration (NIEPA), New Delhi, 110016 India

**Keywords:** Ability to delay gratification, Educational transition, Tracking choice, Vocational education, General education, Cognitive ability & socio-economic status

## Abstract

**Objective:**

A considerable amount of research identified socio-economic status and cognitive ability as robust predictors, the influence of student’s ability to delay gratification (ADG) on their educational transition choice doesn’t received researcher’s attention. To address this gap, the present study examined the incremental power of students ADG in predicting the dichotomous choice i.e. the choice of general or vocational education after successful completion of compulsory schooling.

**Methods:**

Amid Covid-19 pandemic, cross sectional survey via an online mode was found feasible for the data collection process in our study. An online link of survey questionnaire was created in the Google forms and administered to (N = 1024) grade 8 students in the Union Territory of Jammu & Kashmir, India. Multiple binary logistic regressions were conducted to predict the students’ choice, and odds ratios and average marginal effects were reported for better interpretation of results.

**Results:**

Our results showed that students tracking choice differed significantly with respect to their gender and locale (smaller effect), ADG (medium effect), and cognitive ability and socio-economic status (larger effect). The probability of choosing the track of vocational education (with general education track as a baseline category) increases as students ADG decreases, and vice versa. This association of student’s ADG with the choice of vocational education track held same over and above the covariates—socio-economic status, cognitive ability, gender and locale.

**Supplementary Information:**

The online version contains supplementary material available at 10.1186/s40461-022-00134-6.

## Introduction

After successful completion of compulsory schooling, most educational systems around the world offer an important future career choice to their school graduates i.e. either to choose general or to choose vocational track of education (Becker and Glauser 2018; Nießen et al. 2020; Jüttler et al. 2021). The choice of vocational education as a separate track got its momentum because of increase in the demand of vocationally skilled labor force after industrial revolution (Benavot 1983). It was felt that the track of general education has become insufficient to produce vocationally skilled labor force; education systems in different countries were restructured to attract school graduates towards the track of vocational education (Psacharapoulos 1988). However, there was/is a tendency among intellectuals^1^ to distinguish between these two tracks, and often refer this segregation as manual versus mindful, pursuits that are ends in themselves and pursuits that are means to other ends (Bailey and Belfield 2019). Scholars who look primarily through the prism of human capital investment theory posit that skills, whether vocational or general, improve individual’s economic prospectus in comparison to workers without such skills (Becker 1962; Schultz 1961). We see, European countries following the Germany’s dual education system,^2^ put greater emphasis on tracking students towards the path of vocational education at an early stage of their academic life (Powell and Solga 2011). Research fraternity from these countries contends that vocational skills make students productive and facilitates school to work transition at an early age (Solga et al. 2014). However, there is a huge criticism from other group of scholars referring to the sociological theories of class reproduction and social exclusion (Ozer and Perc 2020; Reichelt et al. 2019; Shavit and Müller 2000; Bourdieu 1973). These scholars posit that tracking school graduates, especially students from poor socio-economic families, towards the path of vocational education at an early age limit their chances of attending higher level of education, and as a result also limit their chances of entering high status occupations and professions (Dustmann et al. 2017). Moreover, in the era of Industrial revolution 4.0 (the age of Artificial Intelligence and Machine Learning), the global innovations and developments are so fast that specific vocational skills gained by individuals at their earlier age of life become outdated too quickly (Levin 2012) and as a result these vocationally trained individuals find it difficult to get gainful employment in their later period of life (Levin 2015). Nowadays, the global economy demands the individuals to have higher level of knowledge and intellectual capacity to stay competitive in the twenty-first century (De Fruyt et al. 2015). This may be perhaps one of the reasons why some developed countries (like U.S. Canada, Japan, Norway, Sweden, the United Kingdom) don’t provide too much emphasis on tracking students towards vocational education at earlier age and keep their entire lower secondary school system comprehensive (Hanushek and Woessmann 2006). Research fraternity from these countries contend that general path of education provide students broader knowledge and thorough understanding, and serves as the foundation for life-long learning and on-the-job training (Hanushek et al. 2011).

Keeping in view the fundamental difference discussed above, and the differences in the costs and benefits (individual, social, short term and long term) between the two tracks (e.g., see Hanushek et al. 2011, 2020; Becker and Hecken 2009; Psacharapoulos 1988, 1997 and references therein), the decision on final choice at this stage of academic path is challenging for students (Jüttler et al. 2021). Because such a decision will influence student’s future career goals, wages, income, employment and job satisfaction (Banerjee 2016), and will play a key role in shaping their long-term educational attainment, career prospects and a wide range of related outcomes such as health and well-being (Dustmann et al. 2017). Considering the better status and public image of general path than vocational with regard to social position, employment, occupational outcomes, prestige, income expectations and status preservation (Foster 2002), one might assume that all eligible school graduates would choose the track of general education. However, a wide range of research evidenced that student’s family background (socio-economic status) and previous academic performance (cognitive ability) influence their final tracking decision at this stage (e.g., see Jüttler et al. 2021; Nießen et al. 2020; Usslepp et al. 2020). Usually students from poor socio-economic background families and those with poor academic performance are more likely to choose the vocational path, whereas students from rich (economically, socially and culturally) families and with high cognitive ability are more likely to choose the general path. Given the nature of these two influential factors (students’ socio-economic status and cognitive ability); these are difficult to intervene and change from a policy intervention perspective (Usslepp et al. 2020). Therefore, this is apposite to examine more amenable factors that contribute to successful educational transitions and that could be best targeted by policy intervention and training programs and help students to master educational transitions (Nießen et al. 2020). Students who make unsuccessful transitions are more likely to feel marginalized, are weak in academic performance and more likely to dropout (Coffey 2013). In this context, an important factor identified in the literature which may have a direct association with the student’s tracking choice is the student’s ability to delay gratification. While a considerable amount of empirical studies tested the influence of student’s ability to delay gratification on their different life and educational outcomes (e.g., see Backes-Gellner et al. 2021; Bettinger and Slonim 2007; Bembenutty and Karabenick 1998; Mischel and Ebbesen 1970, Mischel et al. 1989), empirical evidence on influence of student’s ability to delay gratification on their post compulsory school transition choice has not received researcher’s attention and is almost absent in the literature. As a result our understanding on student’s educational tracking choice model remained incomplete. The purpose of this study is to fill this research gap. The rest part of the paper includes the discussion on student’s ability to delay gratification and its linkage with educational transition choice, followed by the discussion on present study investigation and operational model, followed by the methodology including the detailed description on coverage, sample, variables and instruments to measure these, data collection process and analysis, the results, their interpretation and the discussion on findings. At the end, the paper discussed the limitations of present investigation, suggestions for future investigation, and finally conclusive remarks.

## Ability to delay gratification (ADG) and its linkage with student’s educational transition choice

Ability to delay gratification (ADG) refers to individual’s willingness to defer or postpone a small instant pleasure for a larger distant pleasure (Bembenutty et al. 1998). The concept of ADG got its popularity from the famous psychological experiments ‘Marshmallow tests’ conducted by Mischel and Ebbesen (1970) and Mischel et al. (1989). In these experiments, Mischel and his fellow associates put one marshmallow (candy) on the desk in front of each and every kid. The experimenter offered a deal to these kids that I am going to leave the room for fifteen minutes and those who did not eat the candy before my return would be given another candy. However, those who eat the candy before my return would not get a second one. This dichotomous choice offered to students was simple: either to eat one candy right now or to wait for a while and get two candies later on. The CCTV footage of these experiments showed that mouth of maximum kids got watery and ate the candy as soon as Mischel left the room. Some children initially controlled themselves, but in the end gave up. Only a small number of kids managed to control their patience for the entire time. Mischel conducted a series of longitudinal and follow up studies on these kids for more than 40 years and proved that ADG is essential for success in life. The results of these empirical studies showed that kids who waited and showed patience to get the second candy largely determined their success in different educational and life outcomes. Based on these experiments, numerous studies have found that those with higher ability to delay instant pleasure were found better performers in international PISA and SAT tests (e.g., see OECD 2020; Hanushek et al. 2020), have less probability to drink alcohol or smoke (Khwaja et al. 2007), are less emotional and less likely to drop out from school (Sutter et al. 2013; Backes-Gellner et al. 2021), are dedicated and properly plan their activities and goals, are more likely to choose science stream after compulsory schooling (Bembenutty et al. 1998; Mischel et al. 1989). However, those with less ability to delay instant reward were found poor in their educational and labor-market outcomes (Sutter et al. 2013; Cadena and Keys 2015; Kosse and Pfeiffer 2012). While, the direct research on the influence of students ADG on student’s educational transition choice is not available. However, keeping in view the influence of students ADG on different educational and career outcomes, as highlighted in the studies mentioned above, it is reasonable to examine its influence on the student’s educational transition choices. Our reasonability is in line with the studies conducted by numerous researchers (e.g., see Jüttler et al. 2021; Usslepp et al. 2020; Nießen et al. 2020). These researchers, after identifying the influence of personality traits and vocational interests on the student’s different educational and life outcomes (e.g., see references in above three studies mentioned), examined influence of student’s personality traits and vocational interests on their educational transition choices. Here, I also propose that students ADG might be a significant predictor and will help us in understanding the student’s educational transition choice.

## Present investigation

Despite empirical evidence, as mentioned above, on the association between student’s ADG and their different educational and life outcomes, it remained mostly ambiguous whether student’s ADG play a key role in predicting their educational transition choice. To fill this research gap in the existing body of literature, the present study examined the influence of students ADG in predicting their educational transition choice i.e., the dichotomous choice of general or vocational education after successful completion of compulsory schooling. Typically, the track of vocational education develops job ready vocational skills and vocational track students are ready to enter the job market immediately and have earnings instantly, although smaller. Whereas, students who decide to choose the general track of education had to make additional investments in terms of time, effort, costs, and postpone instant earnings against the larger future gains. In view of this trade-off between the two tracks of education, the proposed hypotheses in the present study were formulated accordingly as follows;

### **H1a**

Students with low ADG are more likely to choose vocational education.

### **H1b**

Students with high ADG are less likely to choose vocational education.

Assuming that students educational transition choice is a function of their ADG, the operational model for students’ vocational or general tracking decision was formulated accordingly as;

1 $${\mathbf{Logit}}\left( {{\mathbf{Y}}_{{\mathbf{i}}} } \right) = {{\varvec{\upbeta}}}_{{\mathbf{0}}} + {{\varvec{\upbeta}}}_{{\mathbf{1}}} {\mathbf{X}}_{{\mathbf{i}}} + {\mathbf{y}}_{{\mathbf{i}}} ,$$

where **Yi** is the choice made by an individual student **i** and **Y**_**i**_ = **0** when a student’s choice is vocational track and **Y**_**i**_ = **1** when an individual student chooses a general track. **Logit (Y**_**i**_**)** is the probability/odds ratio of individual ***i*** participating in the track of vocational education compared to the reference category of participation in the track of general education. **β**_**0**_ is an intercept term, **β**_**1**_ is a slope coefficient which will give the coefficient estimate for predictor **X**_**i**_ i.e., students ADG in log-odd units. Since we use logistic regression in this study and accordingly assume that the error term **y**_**i**_ has a standard logistic distribution.

## Methodology

### Coverage and sample

The unit of examination in the present study was the individual grade 8 student from the Union Territory of Jammu and Kashmir (J&K). There are several reasons why I considered J&K as an appropriate location for data collection in the present investigation. First, for students to choose vocational track there must be employment opportunities to which they can look forward to. This would be less prevalent in a small town/village or in a rural district or in a state/union territory where the main source of income is still agriculture and the industrialization level is extremely low. According to the reports of Human Development Index (HDI) (2020), J&K is ranked among the medium and 17th out of the total 36 states and union territories of the India. The HDI of the J&K is about 0.688, 0.001 standard deviation above the national average of 0.645. Economically, agriculture and industry both are the main sources of income for the people of the J&K (Economic Survey 2020–21, https://www.indiabudget.gov.in/economicsurvey). No doubt, the growth of modern industrialization and private sector initiative in J&K is low due to the disturbed political environment (Rangarajan 2010); there are numerous traditional opportunities for the vocational graduates to work in handicraft sector, travel and tourism sector, horticulture, livestock, dairy and poultry, cold storage and food processing, paper mache, carpet, shawl and namdah making, wood carving, manufacturing cricket bats and in various micro, small and medium enterprises (Handicrafts Department, Govt. of J&K, www.jkhandicrafts.com). Another factor that makes J&K favorable location for this study is the broad scale availability of vocational and general schools in the union territory. Within the mainstream education in J&K, there are approx. 600 secondary and senior secondary schools offering general education courses, and among these 353 schools are offering both general and vocational education courses to the successful grade 8 graduates and onwards (UDISE+ 2018; NSSO 2015). Besides, vocational courses are offered in 26 industrial training institutes (ITIs) of the UT. Another important point of consideration here is that our focus was to examine the students intended choices, not the choices already made by them. That is why the present study focused on students currently enrolled in grade 8. After enrolling in grade 9, the tracking decisions are already made by the students, and there is a possibility that these students may try to justify and defend their past decisions while responding to the survey. No doubt, students belonging to higher grades will have a better clarity on tracking decisions, but usually it is at the end of grade 8 or after successful completion of 8 years of schooling in all over India; students for the first time in their academic path face a choice of general and vocational education (see detailed descriptions provided in Kumar et al. 2019; Agarwal 2012, 2014; Agrawal and Agrawal 2017; Banerjee 2016; Aggarwal et al. 2011; Chandrasekhar et al. 2006). Also, the recent policy agenda in India emphasizes on attracting and tracking the larger proportion of students towards the path of vocational education immediately after successful completion of 8 years of schooling (NEP 2020).

While, in the literature, 10 events per variable (EPV of 10) is widely acceptable by the researchers as the lower limit for developing prediction models that predict a binary outcome (Demidenko 2006). However, for analyzing the larger population sizes, researchers recommend a minimum sample size of 500 is essential (Bush 2015). More, EPV of 50 and formula (*n* = 100 + 50*i)* where *i* is the total number of predictors included in the analysis are also suggested by the researchers. Moreover, logistic regression requires large sample sizes because maximum likelihood estimates are less powerful than ordinary least squares used for estimating the unknown parameters in a linear regression model. Keeping in view the coverage of our study and the number of variables included in the analytical model, a sample of (N = 1024) was considered to be a good representative sample. To ensure that the desired sample of students are currently enrolled in grade 8 and in future had an equal access to choose between the two tracks, the survey questionnaire was administered to grade 8 students currently enrolled in 60 identified schools of the UT which (i) at least function from grade 8 and onwards (ii) either offer both general and vocational education courses within their campus or are located close to industrial training institutes of the UT. Schools offering only general education courses were excluded from the analysis on the argument that the choice of vocational education is simply inaccessible to the enrolled students. A complete list of district and school wise distribution of sample students is provided in Additional file 1: Table S1).

### Variables, their operational definitions and measuring instruments

#### Educational transition choice

The dependent/outcome variable in this study was the student’s educational transition choice after successful completion of 8 years of schooling i.e., either to choose general education or to choose vocational education. The choice of general education includes the academic subjects like Languages, Mathematics, Science, Social science, Humanities that prepare students for higher education leading to a university degree (Rose 2008). The choice of vocational education include trades like electrician, plumber, fitter, beauty and wellness, IT and communication, and more similar courses that prepare students for employment after high school graduation by providing them training in specific occupations. The questionnaire asked the students about their intended educational choice after successful completion of grade 8 (see question 5 of the questionnaire). Given the two options—general or vocational education, students were required to mark the one they are likely to choose from after grade 8. Necessary information about the two given options was provided to students before filling out their responses. Those who intended to choose vocational education were coded as (= 0) and those who intended to choose general education were coded as (= 1).

#### Ability to delay gratification (ADG)

The explanatory/independent variable in the present study was student’s ability to delay gratification (ADG). Students ADG refers to their readiness to delay immediately available option/s for the academic opportunities that are provisionally distant but ostensibly worthy (Bembenutty and Karabenick 1998). This ability in students is operationally defined as Academic Delay of Gratification. In this study, student’s ADG was measured by using the most popular and preferred scale i.e., Academic Delay of Gratification Scale (ADOGS) developed by Bembenutty and Karabenick (1998). ADOGS measures student’s ability to delay gratification in academic contexts. This is a 4 point Liket scale with 10 pairs of items. Each pair contains two alternatives. Alternative ‘A’ includes non-academic immediate choices such as watching movies, playing games, going for picnic, not attending lectures etc. Alternative ‘B’ includes delayed academic options such as completing assignments and project work before deadline, using school library and laboratory resources, discussing content with teachers, going thoroughly through course material etc. The four responses for each item were “Definitely choose A”, “Probably choose A, Probably choose B, Definitely choose B” and coded from 1 to 4 respectively. Students were required to choose the one from these 4 response options for each pair of items. The mean score for each student was calculated simply by dividing the total individual score by 10. This mean score provided the measure of ADG for each student. The higher is the mean score between 1 and 4, the greater is the presence of ADG in the student. Cronbach’s Alpha for the original scale is 0.73, Chakraborthy (2017) found (= 0.71) in Indian context and in our study (= 0.72), indicated that the scale is reliable to use in the given context (see Additional file 1: Table S2 for the full scale).

#### Control variables

In order to examine the incremental predictive power of students ADG on their educational tracking choice, four statistical control variables were included in the analysis. These are students (a) socio-economic status (b) cognitive ability (c) gender and (d) locale. Beyond these four covariates, although there are several factors (costs and returns to general and vocational education, unemployment and job growth rate, skill demand in the labor market) influencing the student’s transition choices. These may be best captured in a longitudinal research framework, therefore not included in the present study.

#### Socio-economic status (SES)

Socio-economic status (SES) is one’s position in the social hierarchy and access to resources necessary for healthy and peaceful life (Sirin 2005). Three most common proxy measures of one’s SES in the literature are (1) income (2) occupation and (3) education (Galobardes et al. 2006). In order to avoid the problem of multicollinearity and for better interpretation of the results, researchers mostly prefer and recommend using one proxy measure for SES (Oakes and Rossi 2003). The occupation of parents as a proxy measure of SES was found difficult to incorporate in the present study given that there are hundreds of occupations in even the simplest of taxonomies. For income, studies have highlighted that students, most often, are less aware about their parents income, and there is a tendency of misreporting, where over reporting of parental income is much more common than underreporting (Galobardes et al. 2006). For older individuals (25 years and above), as in our case students parents are, educational attainment is regarded as an appropriate substitute measure of SES. The logic may be that educational attainment for older people remains more or less constant, is easy to measure, and there is a possibility that students may make their responses truthfully. While researchers tend to measure educational achievement by either highest degree earned (e.g., Primary, High School, College, University) or in number of years of schooling (e.g., 1–30) (Sirin 2005), this study used the later. Students were asked to write down their fathers as well mothers education in terms of the total number of years of schooling they have successfully completed. However, in analysis, this study used only the educational achievement of either father or mother, the one with the highest number of schooling years.

#### Cognitive ability (CA)

Cognitive ability (sometimes referred as general intelligence, hereafter CA) is one of the most extensively studied topics within behavioral sciences. It refers to the ability of an individual to think rationally, solve problems, make sense of difficult situations, and learn from previous experiences (Gustafsson and Undheim 1996). In literature, the two appropriate measures of cognitive ability are (1) intelligence tests (IQ tests) (2) standardized academic achievement tests. Usually, achievement tests assess the level of knowledge in academic subjects, whereas, IQ tests assess how fast a student solves the unknown problems (Borghans et al. 2016). However, both these assessment scores were found strongly correlated (Rinderman 2007). In this study, average of total marks percentage obtained by grade 8 students in their previous three grades i.e., in grades 5, 6 and 7, was used as a proxy measure of their cognitive ability. The information on this was directly collected from the school records.

#### Gender and locale

Besides the two established predictors (CA and SES), two more student background characteristics i.e., gender and locale were included in the study as covariates. Both gender and locale were operationalized dichotomously where gender represented biological sex (0: female, 1: male) and locale represented geographical place of living (0: urban, 1: rural).

#### Data collection process

The data required for the present analysis was collected from the sample of students during the period of Covid-19 in the union territory. The pandemic restrictions created a challenging atmosphere in the conduct of research and prevented the research fraternity from employing the conventional methods of data collection (Townsend et al. 2020). In the middle of social distancing and quarantine protocols, an online survey was considered an appropriate technique for data collection in our study (Dodds and Hess 2020). While, keeping in view the digital divide in India (Mathivanan et al. 2021), there is a possibility that online student survey may lead to some bias. However, the current pandemic forced the worldwide education system to shift online, and as a result parents, even from poor families, were forced to manage or buy smart phones or laptops for their children necessary for online education (Sengupta 2022). In our study, all the grade 8 students in 60 identified schools were found participants of their classroom Whatsapp groups. An online link of survey questionnaire was created in the Google forms and shared in their classroom Whatsapp groups only after the approval from their respective school heads. Students were asked to fill all the items of the questionnaire at their own comfort. Necessary help and important information, wherever required, was also provided to the students via these Whatsapp groups to complete the survey with ease. After successive follow-ups, all the students successfully completed the survey, and as a result our survey was free from the usual problem of online surveys i.e., the problem of non-response error.

#### Analysis

Since our outcome is a dichotomous variable i.e., either the choice of general or vocational, the appropriate method for predicting this choice is binary logistic regression (Mood 2010). Given that logistic regression coefficients are difficult to interpret; our study reported Odds Ratios (OR) and Average Marginal Effects (AMEs). First, the study examined the association between student’s ADG and the outcome (Model I). In the second Model, covariates (SES, CA, gender and locale) were added in order to investigate the incremental predictive power of student’s ADG on the outcome over and above these covariates. Finally, in the III to VI model, the study additionally included one covariate at a time i.e., SES in the III model, CA in the IV model, gender in V model and locale in VI model. Before, running the logistic regression analysis in the SPSS software, the study reported the values of Chi-square test to see the association of categories in each of the variables gender (male and female) and locale (rural and urban), Crammer’s V for effect size to see how strongly these categories are associated, t-test to see if there is a statistically significant difference between the means of two groups for students ADG, SES and CA, and Cohen’s d to see the effect size for the comparison between two means (Capraro 2004). Besides, Hosmer–Lemeshow test was used for testing the goodness-of-fit, and three tests (likelihood ratio, score, and Wald tests) for testing the overall model evaluation.^3^

## Results

### Descriptive statistics

Descriptive statistics were used to characterize our sample students in two post compulsory educational transition tracks (see Table 1). 19.8% i.e., approximately one-fifths of the sample students had intended to choose the vocational track and 80.2% i.e., approximately four-fifths intended to choose the general track. Altogether, the proportion of gender of sample students was approximately equal (Male = 51.9%, Female = 48.1%), but it dispersed significantly between the two outcome choices (see Table 2). While the choice of general track was largely intended by female students (58.4%), the choice of vocational track was largely intended by male students (61.2%). In case of locale, the overall proportion of respondents from rural and urban areas was more or less equal (Rural = 53.6%, Urban = 46.4%). However, the locale variations between the two outcome choices were even larger than those for gender (see Table 3). While the choice of general track was largely intended by urban students (61.3%), the choice of vocational track of was largely intended by rural students (63.5%).

**Table 1:** Final sample description

	N	%	Male (%)	Female (%)	Rural (%)	Urban (%)			
General	821	80.2	41.6	58.4	38.7	61.3			
Vocational	203	19.8	61.2	38.8	63.5	36.5			
Total	1024	100	51.9	48.1	53.6	46.4			

Variables	Overall		General		Vocational		Min.	Max.	Items
	M	SD	M	SD	M	SD			
ADG	2.91	8.61	3.11	9.03	1.73	4.29	10	40	10
SES	8.22	2.11	12.35	3.96	5.31	2.86	0	25	1
CA	56.41	19.20	62.09	12.47	43.21	14.73	33	100	1

*N* number of students, *M* mean, *SD* standard deviation, *ADG* ability to delay gratification, *SES* socio-economic status, *CA* cognitive ability

**Table 2:** Chi-square tests (gender)

	N	Value	df	Crammer’s V	Asymp.Sig (2-tailed)
Pearson’s Chi-square	1024	31.816^a^	1	0.191	0.001

^a^0 cells (0.0%) have expected count less than 5. The minimum expected count is 39.54

**Table 3:** Chi-square tests (locale)

	N	Value	df	Crammer’s V	Asymp.Sig (2-tailed)
Pearson’s Chi-square	1024	79.541^a^	1	0.313	0.000

^a^0 cells (0.0%) have expected count less than 5. The minimum expected count is 47.11

The average values of student’s ADG for general and vocational track students gave a signal of a medium effect (Cohen’s d = 0.05) of student’s ADG (see Table 4). While, the students who intended to choose general track of education had the higher ADG with a mean value of 3.11, students in vocational track had a mean value of 1.73. The average values of the parental education of general and vocational track students gave a signal of a stronger effect (Cohen’s d = 0.07) of parental socio-economic status (see Additional file 1: Table S3). While, the students who intended to choose general track of education had the higher family socio-economic status with a mean value of 12.35 years of parental education, students in vocational track had a mean value of 5.31 years. Finally, the average values of the student’s previous academic performance also gave a signal of stronger effect (Cohen’s d = 0.08) of student’s cognitive ability on the outcome variable (see Additional file 1: Table S4). While the students in general track had the highest academic percentage marks with an average of 62.09%, students in vocational track had 43.21%. The above results signal that students intended choice for general and vocational track of education disperse significantly in relation to their gender and locale (smaller effect), ADG (medium effect), and socio-economic status and cognitive ability (stronger effect).

**Table 4:** Independent sample t-test (ADG)

	Levene’s test		t-test for equality of means					
	F	Sig	T	Df	Sig. (2 tailed)	Mean difference	Std. error difference	95% CI of difference
ADG equal variances assumed	10.221	0.000	3.119	1022	0.002	1.038	0.019	[0.271, 2.991]
Equal variances not assumed			5.779	991.846	0.001	1.038	0.012	[0.272, 3.011]

Finally, the study reported three inferential statistical tests (the likelihood ratio, score, and Wald tests) for testing the overall model evaluation, and Hosmer–Lemeshow test for testing the goodness-of-fit (see Table 5). The results from all the three tests show that the analytical model with predictor variables is more effective than the null model. Hosmer–Lemeshow test result (= 8.27) is insignificant reflecting that the model is fit to the data well. Missed data calculated with the Full Information Maximum Likelihood method and didn’t affect the results too much as it ranged from 0.6 to 6.1% per variable in the analysis.

**Table 5:** Overall model evaluation and goodness-of-fit statistics

Test	Categories	*χ*^2^	p
Overall model evaluation	Likelihood test	14.37	0.03
	Score test	11.71	0.01
	Wald test	11.01	0.02
Goodness-of-fit-test	Hosmer and Lemeshow test	8.27	0.46

**Table 6:** Model 1 **(**predicting intended track choice with a model including student’s ADG only)

Variable	Model I		Model II	
	OR [95% CI]	AME [95% CI]	OR [95% CI]	AME [95% CI]
ADG	0.77* [0.59, 1.04]	− 0.09* [− 0.21, 0.00]	0.54* [0.61, 1.01]	− 0.08* [− 0.20, 0.00]
SES			0.86** [0.76, 0.98]	− 0.13** [− 0.20, − 0.02]
CA			0.97** [0.67, 1.44]	− 0.21** [− 0.30, − 0.05]
Gender			0.69* [0.51, 0.91]	− 0.05* [− 0.12, 0.00]
Locale			0.58* [0.41, 0.76]	− 0.04* [− 0.10, 0.00]
Pseudo R^2^	0.11***		0.51***	

Model 2 (predicting intended track choice with a model including student’s ADG, SES, CA, gender and locale)

[95% CI]: 95% confidence interval; OR: odds ratio; AME: average marginal effect; ADG: ability to delay gratification; SES: socio-economic status; CA: cognitive ability

*p < 0.05, **p < 0.01; ***p < 0.001

### Outcome predictions

The results of the multiple binary logistic regressions used in analysis are reported in the models from I to VI. Results from all of our models clearly show that student’s ADG is a significant predictor of the educational transition choice after grade 8. Model I shows the negative association of students ADG with the choice of vocational track (with general track as a baseline), and explains 11% of variance (see Table 6). As student’s ADG increases by one unit, odds of choosing vocational track are lower (OR = 0.77), and the probability of choosing vocational track decreases by 9%. After controlling for all of our covariates (SES, CA, gender and locale) in Model II, student’s ADG shows negative association with the choice of vocational track, and explains 51% of variance indicating that all of our explanatory variables are significant predictors. In Model II, as student’s ADG increases by one unit, odds of choosing vocational track are lower (OR = 0.54), and the probability of choosing vocational track decreases by 8%. This association of student’s ADG held’s true in all of our remaining models in which covariates were included one at a time (for details, see Additional file 1: Tables S5–S8). After controlling for student’s CA in Model III and SES in Model IV, these two models additionally explained approximately 19% and 17% of the variance respectively. AMEs in all our models indicated that the influence of SES and CA were relatively large. This influence of SES and CA remained largely the same even when student’s ADG and other covariates were included in our models. After controlling for gender (male as a reference category) in Model V and locale (rural as a reference category) in Model VI, these two models additionally explain a small variance i.e., approximately 3.5% and 1.9% respectively. Overall, our results were in line with our expectations and supported our hypothesis.

## Discussion

This study examined the influence of student’s ADG on their educational track choice at the end of grade 8 in the UT of J&K, India. Specially, focusing on the dichotomous choice of general and vocational track of education, our results show significant differences in the student’s ADG between the two tracking choices. Our propositions were supported by our results. Further, our results identified that student’s ADG incrementally predicted the outcome choice over and above student’s CA, SES, gender, and locale. The control variables have additionally increased the overall variance in the outcome depicting the significant influence of student’s ADG over and above these control variables. Among all the predictors in the analysis, student’s CA and SES proved to be the most powerful. Our results over scored the relation of educational transition choice with the CA of students as well as with their SES. These associations are in line with the plethora of previous empirical studies that examined the influence of SES and CA on the educational transition choices (e.g. see Becker and Hecken 2009; Becker and Glauser 2018). Here, this is important to take into consideration that the transition period examined in our study took place relatively earlier in students’ educational academic path (students were on average 13 to 14 years old) when parental SES and student’s CA become more influential for the student’s educational decision than the other factors. However, SES and CA may play less important influence than other factors during later educational transitions (e.g., see Jüttler et al. 2021; Nießen et al. 2020; Usslepp et al. 2020). Although, gender and locale had showed additive relationship with transition success, but the strength of this relation was found weak. Being a female lowered the odds of choosing the vocational track. This influence in the literature has been explained by the certainty that females have relatively low physical strength than males, as a result are less likely to choose labor intensive occupations (Pitt et al. 2012; Round et al. 1999). Because the vocational track of education usually leads to the jobs that are more labor intensive than the general track, so it is relatively less attractive to women than men. Being an urban lowered the odds of choosing the track of vocational education. This influence may be perhaps because of the fact that urban people have more access to take part in higher education preparation activities like visiting the university and have sufficient knowledge about different opportunities in future life. These issues increase the aspirations of urban people for higher levels of education and are keener to enter the university and highly prestigious occupations. Whereas rural people who remain in close proximity to their parents and villages tend to enter the world of work immediately at the early age (Griffin et al. 2011). Overall, our results prove that at this stage of academic path, the choice for general or vocational is not only influenced by student’s cognitive ability, their parental socio-economic status, gender and locale but student’s ADG plays a key role too.

## Limitations and future

This study suffers from several limitations those could be possibilities for conducting the future research. First, our results are helpful in understanding the main effects i.e., student’s ADG has an incremental linear relation with their educational transition success over and above the covariates included. It could be interesting to look into the interaction associations i.e., whether student’s ADG would moderate the associations between established predictors of transition success—SES and CA—and for individual background characteristics—gender and locale. Second, our findings and the associations found cannot be interpreted as causal because of the pseudo influence led by unnoticeable third variables and due to the cross-sectional design of the analyses. A causal association could be best served by experimental or quasi-experimental research. Third, is about the coverage of the study. Since, the respondents were selected from the UT of J&K, India; therefore, given the distinct geopolitical structure of the UT, the external validity and generalizability of our results is limited to J&K. If the differences between the two tracks are exchangeable between the different educational systems, and if the two education systems are comparable, the results may be close to as in our study. However, more empirical evidences are necessary to replicate and expand these findings and generalize them to other systems. Fourth, the data was collected from those students who were currently enrolled in grade 8 and intended to choose between the two tracking choices after successful graduation. However, there is a possibility that actual choices may differ from their intended choices. So this could be interesting if their actual choices could be analyzed at the time of actual transition. Fifth, whilst there are several factors influencing the students educational transition choices such as costs and returns to two tracks, employment opportunities, student’s aspirations and interests, labor market requirements and several more. A comprehensive understanding of student’s educational choice might be an interesting research work. All in all, more empirical evidence is essential to investigate all the important dimensions that contribute to the successful educational transition of students.

## Conclusion

After successful completion of compulsory schooling, an important future career decision that students face in their academic path is the choice of general or vocational education. This study contributes to our understanding by identifying student’s ADG as a significant predictor of their educational transition choice. Even after controlling for SES, CA, gender and locale, our results suggest a direct link between students ADG and their educational transition choice. Among the variables included in our regression models, SES and CA proved to be highly influential factors, followed by the student’s ADG. The other variables (gender and locale) showed additive, although smaller, associations with the tracking choices. Our results suggest that future empirical studies on educational transition might do well by considering student’s ADG in addition to other predictors. At this crucial stage of academic life, our results recommend that there is a need for educational guidance and career counseling intervention and training programs to enhance students ADG. Enhancing student’s ADG might help them to choose an appropriate educational track for the amelioration of their future career success. More specifically, such intervention and training programs might help students from poor families who usually tend have low ADG, and ultimately would promote the equality of educational opportunity.

## Supplementary Information

**Additional file 1: Table S1.** District and school wise distribution of participants in an online survey. **Table S2.** Online questionnaire for students. **Table S3.** Independent sample t-test (socio-economic status). **Table S4.** Independent sample t-test (cognitive ability). **Table S5.** (Model III) predicting intended track choice with a model including student’s ADG and SES. **Table S6.** (Model IV) predicting intended track choice with a model including student’s ADG and CA. **Table S7.** (Model V) predicting intended track choice with a model including student’s ADG and gender. **Table S8.** (Model VI) predicting intended track choice with a model including student’s ADG and locale.

## Footnotes

1 Both Plato in ‘Republic’ and Aristotle in ‘Politics’ posit that vocational people are incapable and there is no element of virtue in vocational occupations (for more details, e.g., see Rose 2008).

2 Dual system of VET (school-based education along with on-the-job training) has received much global attention for its effectiveness in addressing the big issue of youth unemployment in European nations (for more details, see e.g. Powell and Solga 2011).

3 Logistic regression usually does not demand most of the basic assumptions of linear regression—especially the linear association between the outcome and predictors, normality of the error term, and homoscedasticity/heterocedasticity (for more details see Mood 2010).
