# Supplementary material for: Influence of student’s ability to delay gratification on their educational transition choice
Source: Empir Res Vocat Educ Train. 2022 May 13;14(1):6. doi: 10.1186/s40461-022-00134-6 (PMC9100297; doi:10.1186/s40461-022-00134-6)
Supplement: Supplementary file 1 — Additional file 1: Table S1. District and school wise distribution of participants in an online survey. Table S2. Online questionnaire for students. Table S3. Independent sample t-test (socio-economic status). Table S4. Independent sample t-test (cognitive ability). Table S5. (Model III) predicting intended track choice with a model including student’s ADG and SES. Table S6. (Model IV) predicting intended track choice with a model including student’s ADG and CA. Table S7. (Model V) predicting intended track choice with a model including student’s ADG and gender. Table S8. (Model VI) predicting intended track choice with a model including student’s ADG and locale. [file 40461_2022_134_MOESM1_ESM.docx]

**Additional file 1**

**Table S1:** District and school wise distribution of participants in an online survey

| **District** | **School** | **Participants** | **District** | **School** | **Participants** |
| --- | --- | --- | --- | --- | --- |
| 1. Anantnag (271 participants) | GHSS Batkote  GHSS Bijbehara  HSS Soaf  GHSS Doru  GHSS Larnu  HSS Nanil  HSS Shangus  BHSS Sirgufwara  HSS Lessu  BHSS Khiram  HSS Lalan  HSS Mantpora  GHSS Chaklipora  HSS Qamer  HSS Guridraman | 19  16  17  19  21  14  20  24  13  12  17  18  21  17  23 | 2.Pulwama (258 participants) | GHSS Litter  GHSS Kakapora  GHSS Pampore  BHSS Pampore  HSS Pinglena  BHSS Arihal  HSS Hariparigam  BHSS Chersoo  HSS Batagund  GHS Ladhoo  BHS Samboora  GHSS Abhama  HSS Nehama  HSS Achan  HSS Chakura | 20  14  23  21  19  18  12  17  21  18  17  11  10  23  14 |
| 3.Srinagar (240 participants) | GHSS Sonwar  GHSS Nishat  GHSS Soura  GHSS Nawakadel  BHSS Soura  BHSS Khimber  GHSS Khaniyar  BHS Natipora  HS Barzulla  BHSS Gulabagh  GHS Balhama  GHS Zewan  BHS Sonwar  BHS Lasjan  GHS Lasjan | 24  17  20  12  17  19  23  10  12  11  13  17  12  11  22 | 4.Budgam (255 participants) | GHS Shogapora  BHS Humhama  GHS Narbal  HSS Bugam  BHS Pakherpora  HSS Kandora  HSS Otligam  HSS Bemina  HS Dragger  HSS Wadwan  BHS Shogapora  BHS Ompora  GHS Ompora  HS Chanapora  BHS Sogam | 10  19  13  18  17  19  14  22  19  20  21  14  11  19  19 |

**Table S2:** Online Questionnaire for Students

There are two parts in this questionnaire. Part I is to know some of your background characteristics. Part II is to assess the current status of your Ability to delay gratification. Please answer all of the questions. Listen to the explanation from the researcher, wherever required, before providing the answers.

**Part I: Background Information**

1. Gender? a) Male b) Female

1. Locale? a) Rural b) Urban
2. Total marks obtained in the annual examination?
3. Grade V __________________
4. Grade VI __________________
5. Grade VII __________________
6. What is your parents’ (Father’s as well as Mother’s) highest educational qualification?
7. Father ___________________
8. Mother ___________________
9. Which educational track you prefer to enroll in after grade 8?
10. General education b) Vocational education

**Part II**

Please read each set of statements carefully, and tell which course of action you would be more likely to choose and the strength of that choice. Do this by placing a tick mark in front of that choice using the scale below:

**Definitely choose A (=1), probably choose A (=2), probably choose B (=3) and definitely choose B (4)**

| **S. No.** | **Which situation you prefer (A or B)** | | **How strongly you prefer** | | | |
| --- | --- | --- | --- | --- | --- | --- |
|  |  |  | **1** | **2** | **3** | **4** |
| 1 | A | Go to a favorite concert, play, or sporting event and study less even though it may mean getting a lower grade on an exam you will take tomorrow |  |  |  |  |
|  | B | Stay home and study to increase your chances of getting a higher grade |  |  |  |  |
| 2 | A | Study a little every day for an exam and spend less time with your friends |  |  |  |  |
|  | B | Spend more time with your friends and cram just before the test. |  |  |  |  |
| 3 | A | Miss several classes to accept an invitation for a very interesting trip |  |  |  |  |
|  | B | Delay going on the trip until the exam is over |  |  |  |  |
| 4 | A | Go to a party the night before a test and study only if you have time |  |  |  |  |
|  | B | Study first and party only if you have time |  |  |  |  |
| 5 | A | Spend most of your time studying just the interesting material even though it may mean not doing so well, |  |  |  |  |
|  | B | Study all the material that is assigned to increase your chances of doing well |  |  |  |  |
| 6 | A | Skip the class when the weather is nice and try to get the notes from somebody later, |  |  |  |  |
|  | B | Attend classes to make certain that you don’t miss something even though the weather is nice outside |  |  |  |  |
| 7 | A | Stay in the library to make certain that you finish an assignment that is due the next day |  |  |  |  |
|  | B | Leave to have fun with your friends and try to complete it when you get home later that night |  |  |  |  |
| 8 | A | Study in a place with a lot of pleasant distractions |  |  |  |  |
|  | B | B. Study in a place where there are fewer distractions to increase the likelihood that you will learn the material |  |  |  |  |
| 9 | A | Leave right after class to do something you like even though it means possibly not understanding that material for the exam |  |  |  |  |
|  | B | Stay after class to ask your instructor to clarify some material for an exam that you do not understand |  |  |  |  |
| 10 | A | Select an instructor who is fun even though he/she does not do a good job covering the course material |  |  |  |  |
|  | B | Select an instructor for who is not as much fun but who does a good job covering the course material |  |  |  |  |

**Table S3:** Independent sample t-test (Socio-economic status)

|  | **Levene’s Test** | | **t-test for Equality of Means** | | | | | |
| --- | --- | --- | --- | --- | --- | --- | --- | --- |
|  | **F** | **Sig.** | **t** | **df** | **Sig. (2 tailed)** | **Mean Difference** | **Std. Error Difference** | **95 % CI of difference** |
| SES Equal variances  assumed  Equal variances  not assumed | 63.011 | .000 | 8.019  11.779 | 1022  991.846 | .000  .002 | 7.116  7.116 | .441  .441 | [1.176, 13.550]  [1.177, 13.551] |

**Table S4:** Independent sample t-test (Cognitive ability)

|  | **Levene’s Test** | | **t-test for Equality of Means** | | | | | |
| --- | --- | --- | --- | --- | --- | --- | --- | --- |
|  | **F** | **Sig.** | **t** | **df** | **Sig. (2 tailed)** | **Mean Difference** | **Std. Error Difference** | **95% CI of difference** |
| CA Equal variances  assumed  Equal variances  not assumed | 201.771 | .001 | 14.791  19.779 | 1022  991.846 | .000  .002 | 19.24  19.24 | .519  .600 | [12.428, 23.072]  [12.427, 23.073] |

**Table S5: (Model III)** Predicting Intended Track Choice with a Model including student’s ADG and SES

**Variable OR AME**

**[95%CI] [95%CI]**

ADG .75* −.07*

[.58, 1.01] [−.18, .00]

SES .85** − .11**

[.74, .96] [−.20; −.02]

Pseudo R² 0.19***

*Abbreviations: [95% CI], 95% Confidence Interval; OR, odds ratio; AME, average marginal effect; ADG, ability to delay gratification; SES, Socio-economic status; *p < .05, **p < .01; ***p < .001.*

**Table S6: (Model IV)** Predicting Intended Track Choice with a Model including student’s ADG and CA

**Variable OR AME**

**[95%CI] [95%CI]**

ADG .76* −.08*

[.60, 1.01] [−17, .00]

CA .94* −.19*

[.65, 1.40] [−.32, −.09]

Pseudo R² 0.17***

*Abbreviations: [95% CI], 95% Confidence Interval; OR, odds ratio; AME, average marginal effect; ADG, ability to delay gratification; CA, Cognitive ability; *p < .05, **p < .01; ***p < .001.*

**Table S7: (Model V)** Predicting Intended Track Choice with a Model including student’s ADG and Gender

**Variable OR AME**

**[95%CI] [95%CI]**

ADG .81* −.09*

[.55, 1.09] [−16, .00]

Gender .71* −.05*

[.54, .93] [−.12, .00]

Pseudo R² 0.035***

*Abbreviations: [95% CI], 95% Confidence Interval; OR, odds ratio; AME, average marginal effect; ADG, ability to delay gratification; *p < .05, **p < .01; ***p < .001.*

**Table S8: (Model VI)** Predicting Intended Track Choice with a Model including student’s ADG and Locale

**Variable OR AME**

**[95%CI] [95%CI]**

ADG .82* −.11*

[.53, 1.04] [−19, .00]

Locale .61* −.06*

[.41, .86] [−.12, .00]

Pseudo R² 0.019***

*Abbreviations: [95% CI], 95% Confidence Interval; OR, odds ratio; AME, average marginal effect; ADG, ability to delay gratification; *p < .05, **p < .01; ***p < .001.*
